# Supplementary material for: Antitumoral Activity of a CDK9 PROTAC Compound in HER2-Positive Breast Cancer
Source: Int J Mol Sci. 2022 May 13;23(10):5476. doi: 10.3390/ijms23105476 (PMC9146359; doi:10.3390/ijms23105476)
Supplement: Supplementary file 1 [file ijms-23-05476-s001.zip › Supplementary Figure S1.pdf]

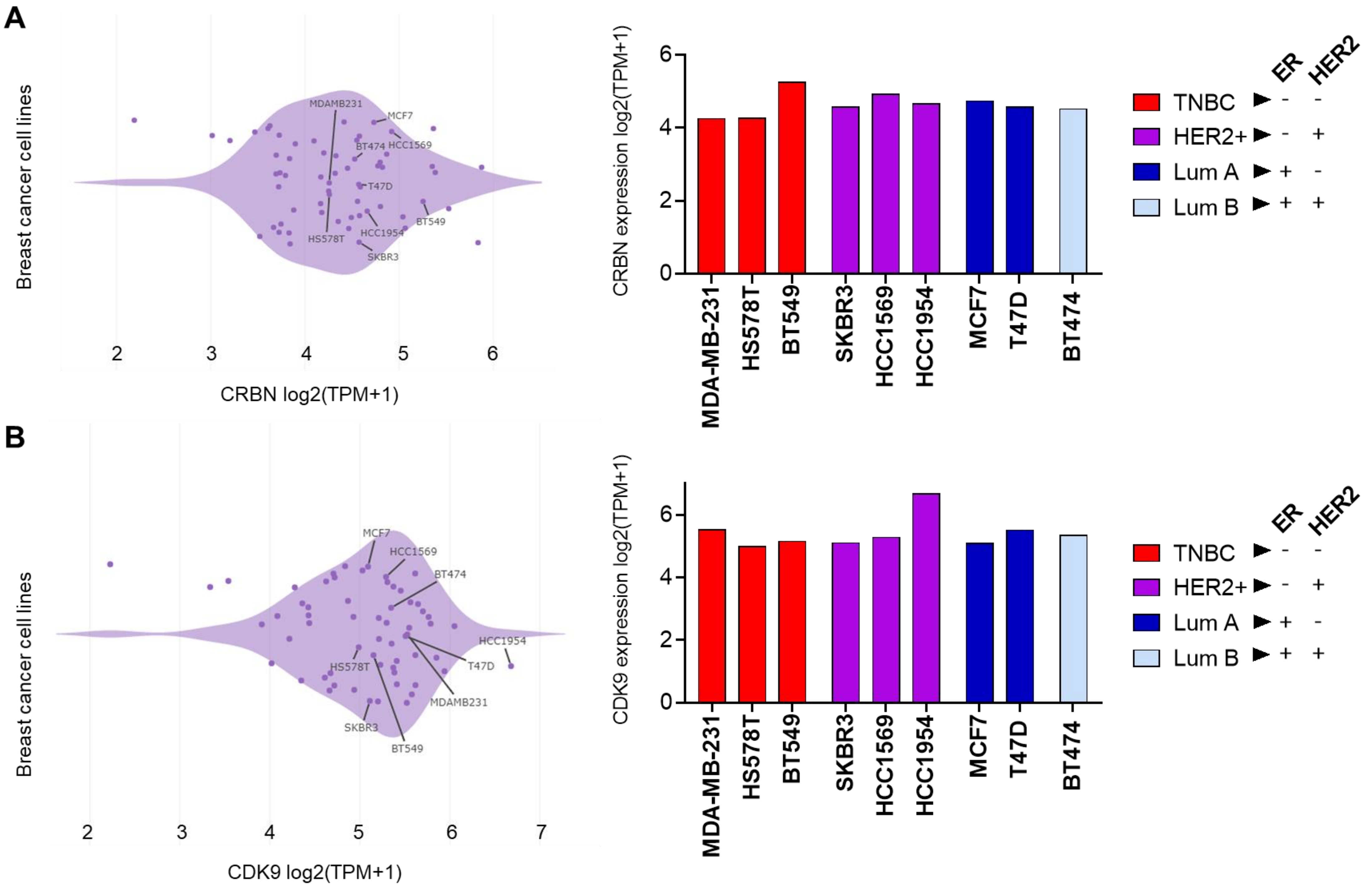
A
Breast cancer cell lines
CRBN log2(TPM+1)
MDAMB231
MCF7
BT474
HCC1569
T47D
BT549
HCC1954
HS578T
SKBR3
CRBN expression log2(TPM+1)
MDA-MB-231
HS578T
BT549
SKBR3
HCC1569
HCC1954
MCF7
T47D
BT474
ER
HER2
TNBC - -
HER2+ - +
Lum A + -
Lum B + +
B
Breast cancer cell lines
CDK9 log2(TPM+1)
MCF7
HCC1569
BT474
HCC1954
T47D
HS578T
MDAMB231
SKBR3
BT549
CDK9 expression log2(TPM+1)
MDA-MB-231
HS578T
BT549
SKBR3
HCC1569
HCC1954
MCF7
T47D
BT474
ER
HER2
TNBC - -
HER2+ - +
Lum A + -
Lum B + +
