# Supplementary figures and images for: Antitumoral Activity of a CDK9 PROTAC Compound in HER2-Positive Breast Cancer
Source: Int J Mol Sci. 2022 May 13;23(10):5476. doi: 10.3390/ijms23105476 (PMC9146359; doi:10.3390/ijms23105476)

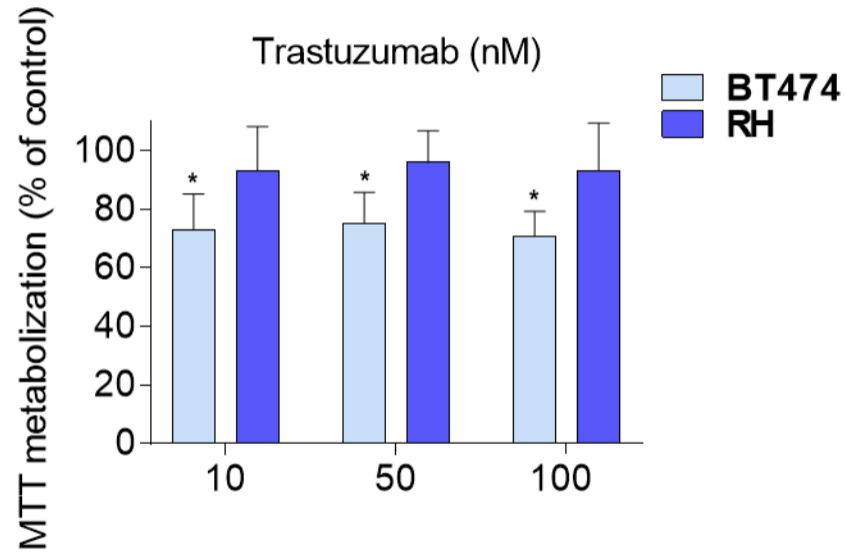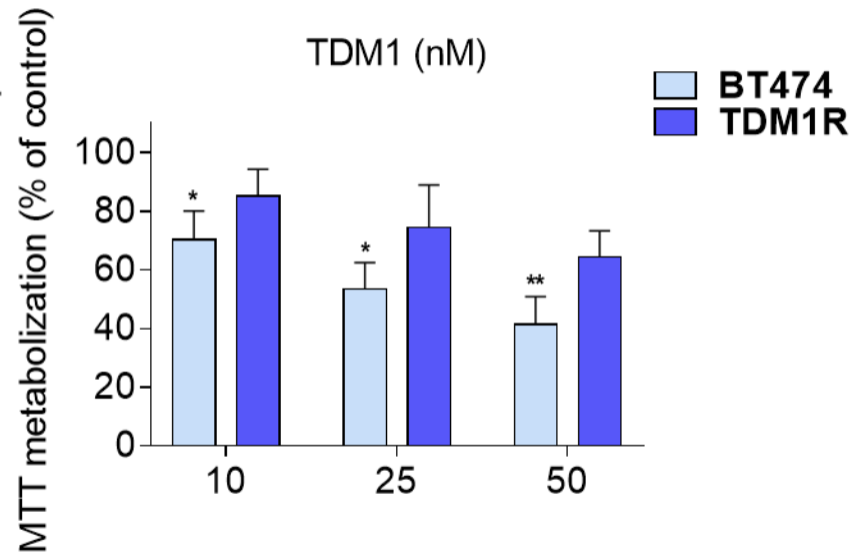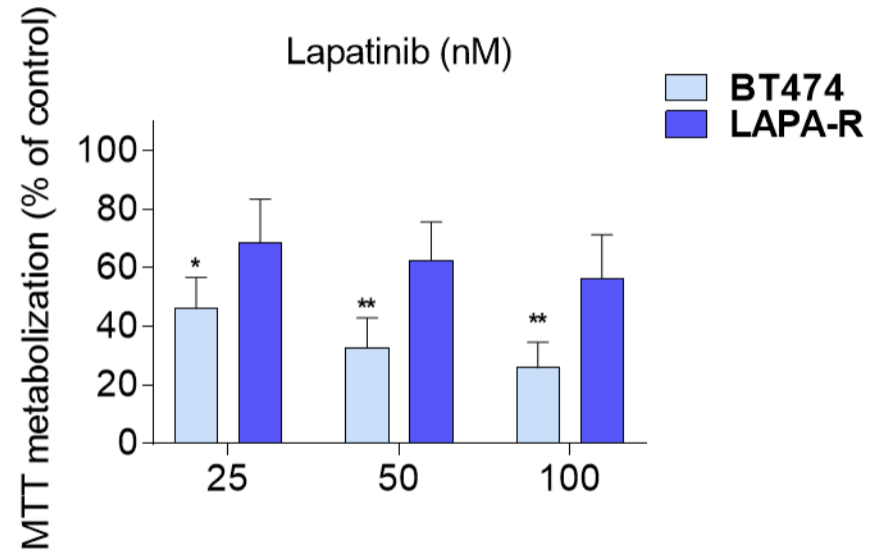

Supplement: Supplementary file 1 [file ijms-23-05476-s001.zip › Supplementary Figure S2.pdf]

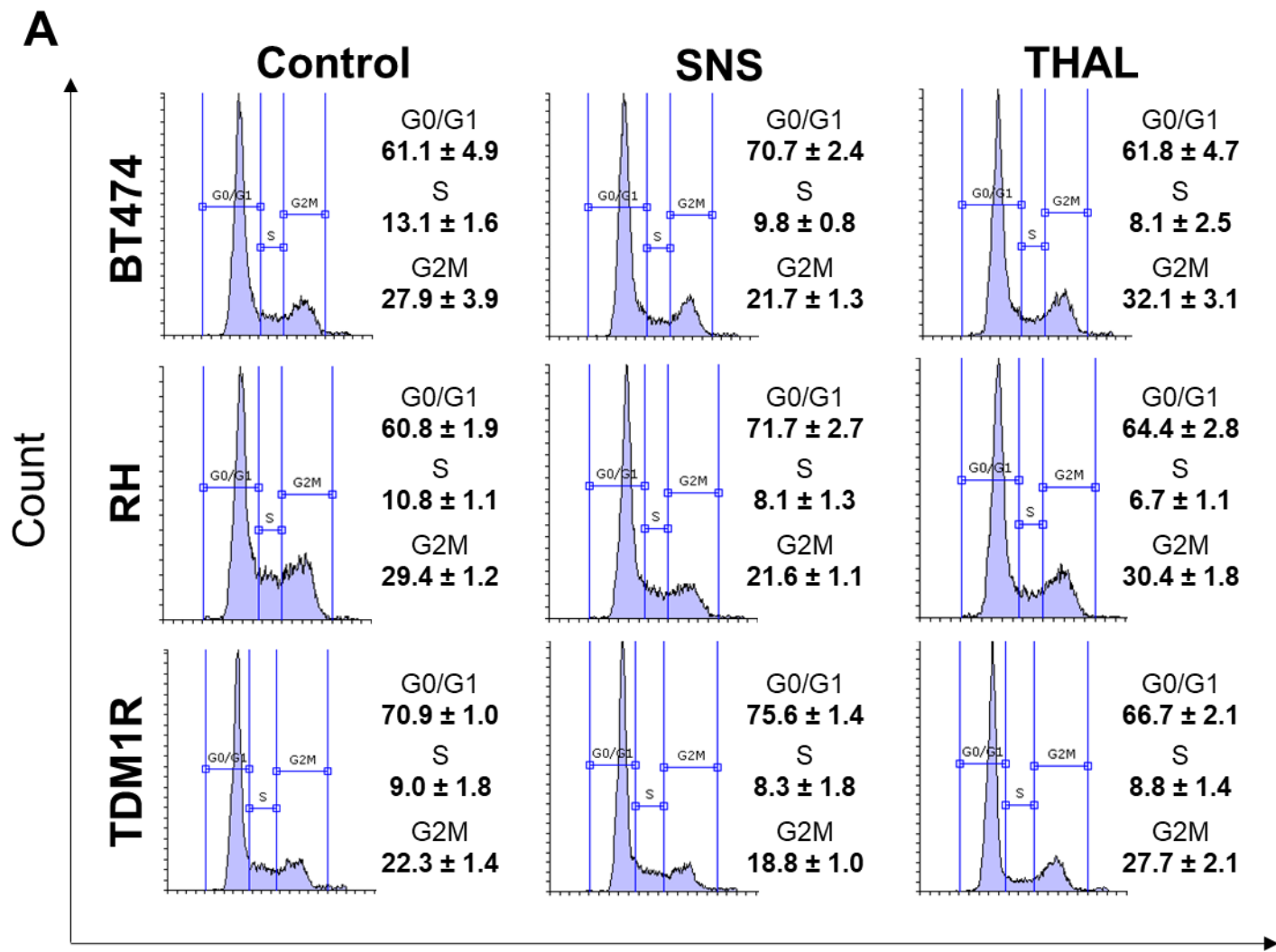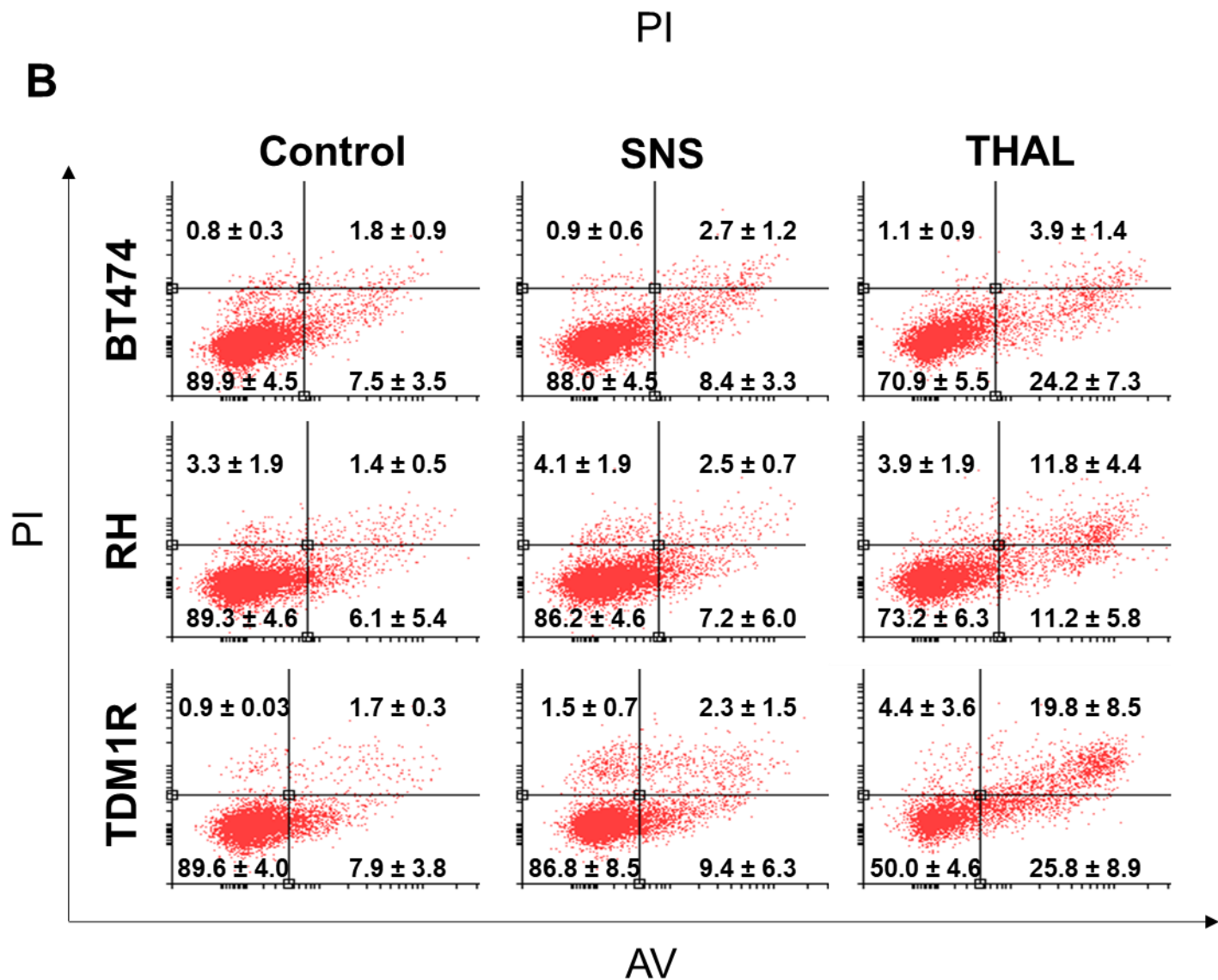

Supplement: Supplementary file 1 [file ijms-23-05476-s001.zip › Supplementary Figure S3.pdf]

**A**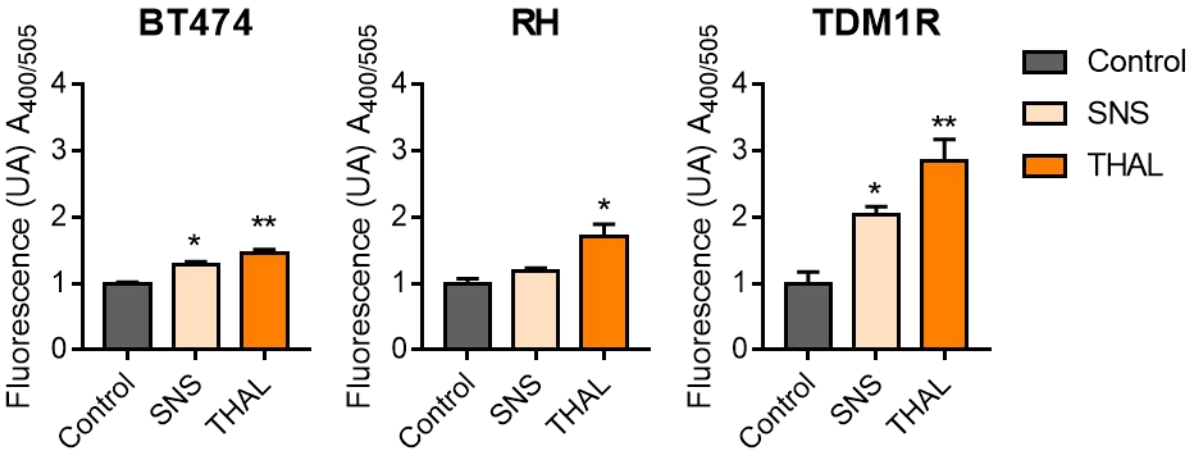**B**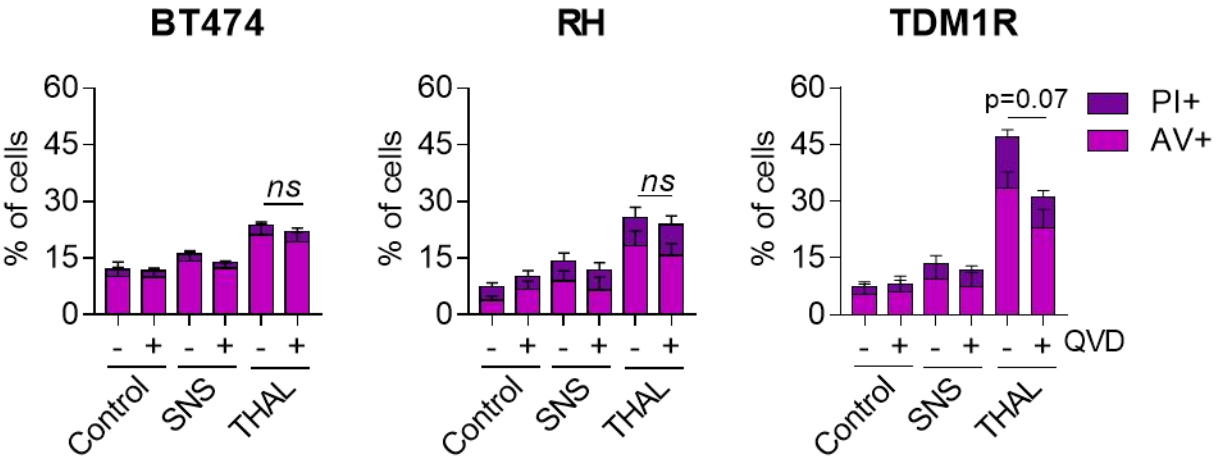**C**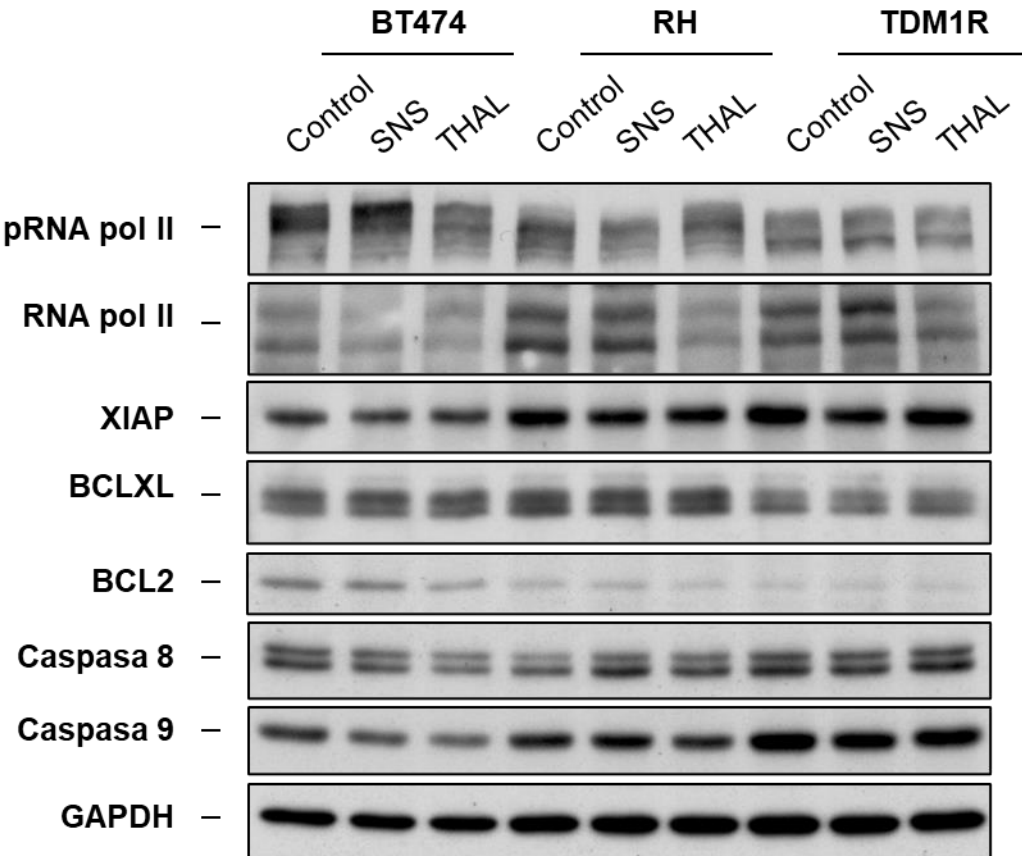

Supplement: Supplementary file 1 [file ijms-23-05476-s001.zip › Supplementary Figure S4.pdf]

**A**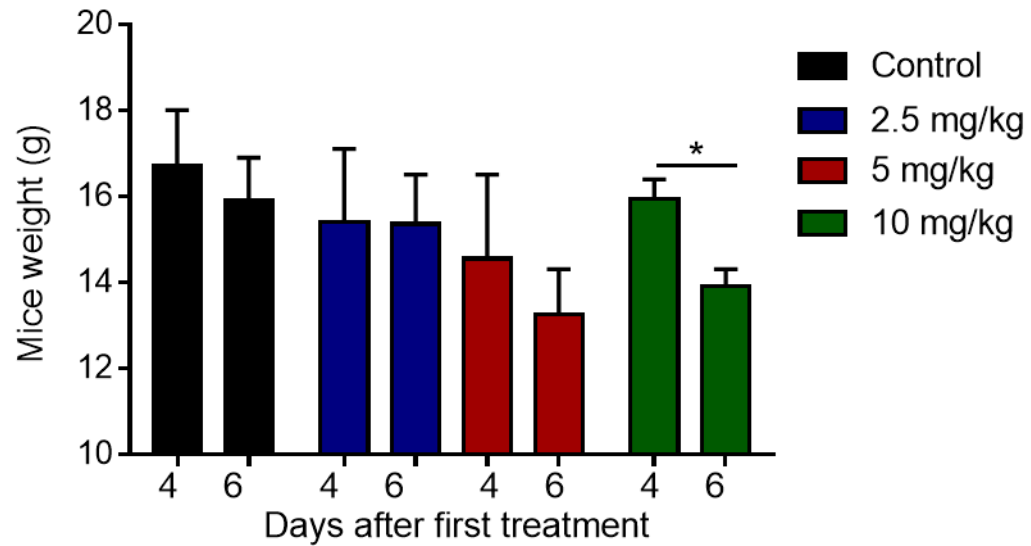**B**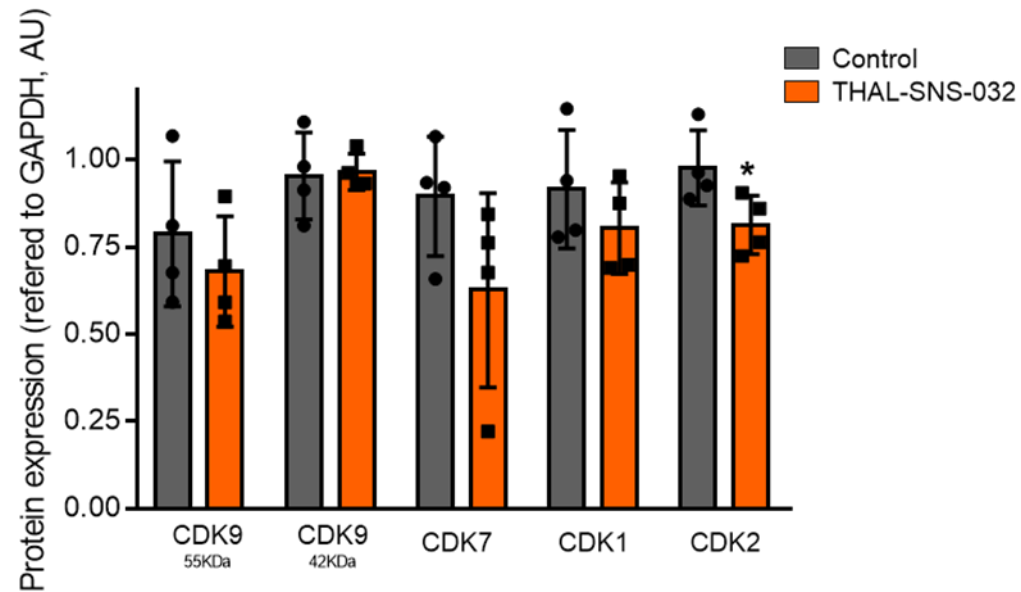

Supplement: Supplementary file 1 [file ijms-23-05476-s001.zip › Supplementary Figure S5.pdf]

**A**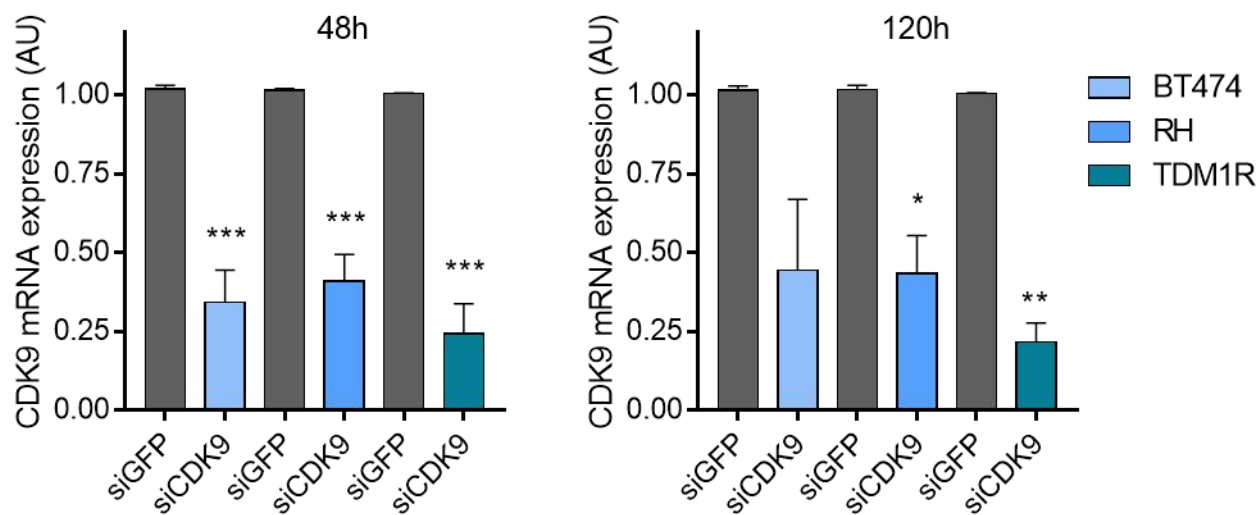**B**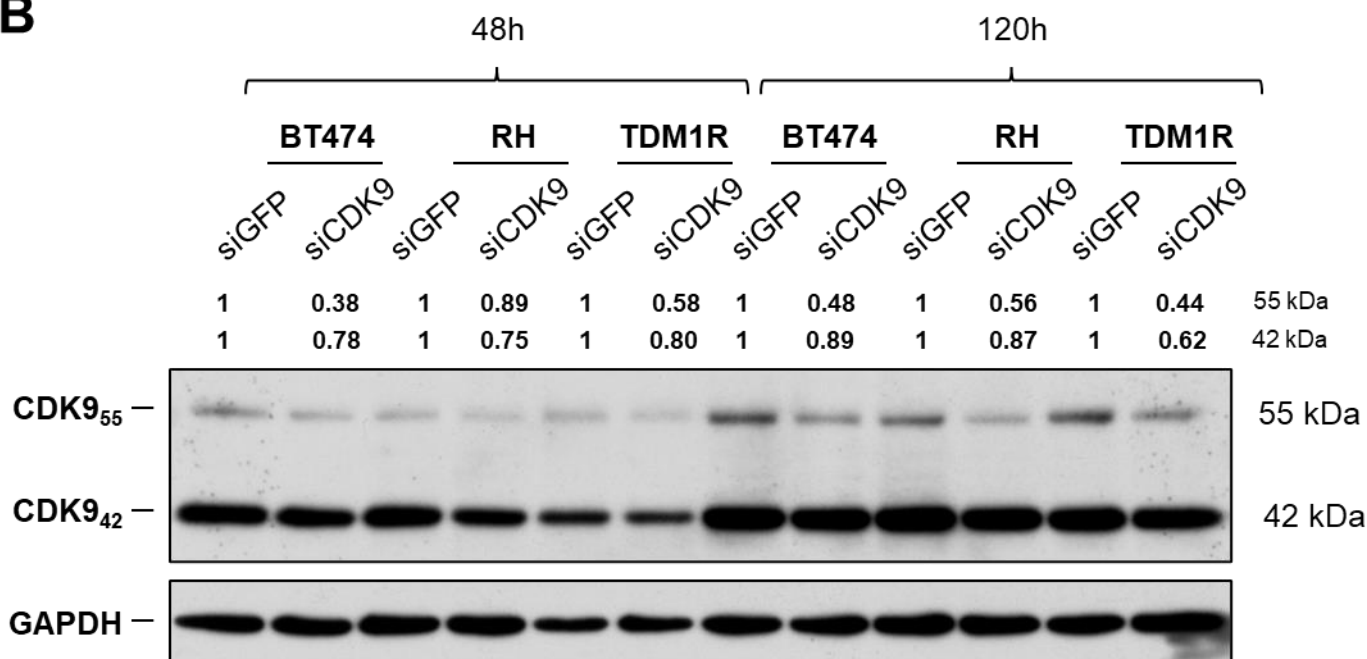**C**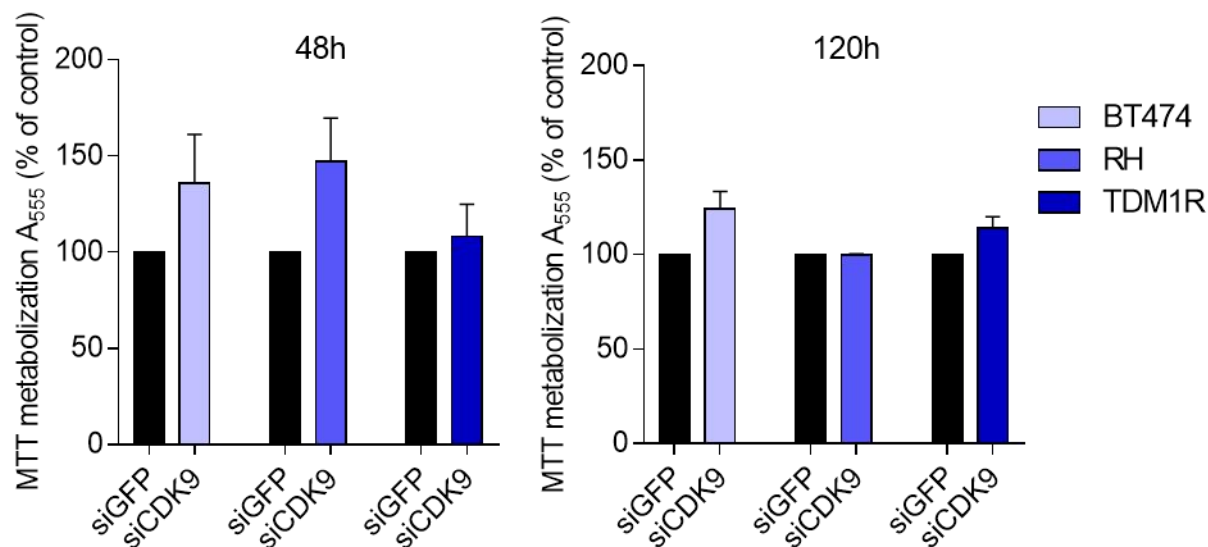

Supplement: Supplementary file 1 [file ijms-23-05476-s001.zip › Supplementary Figure S6.pdf]
